# A missense mutation separates distinct functions of the Zic-family transcription factor REF-2

**DOI:** 10.17912/micropub.biology.000232

**Published:** 2020-03-16

**Authors:** Michael P. Hart, Oliver Hobert

**Affiliations:** 1 Department of Genetics, Perelman School of Medicine, University of Pennsylvania, Philadelphia, USA; 2 Columbia University, Howard Hughes Medical Institute

**Figure 1:**
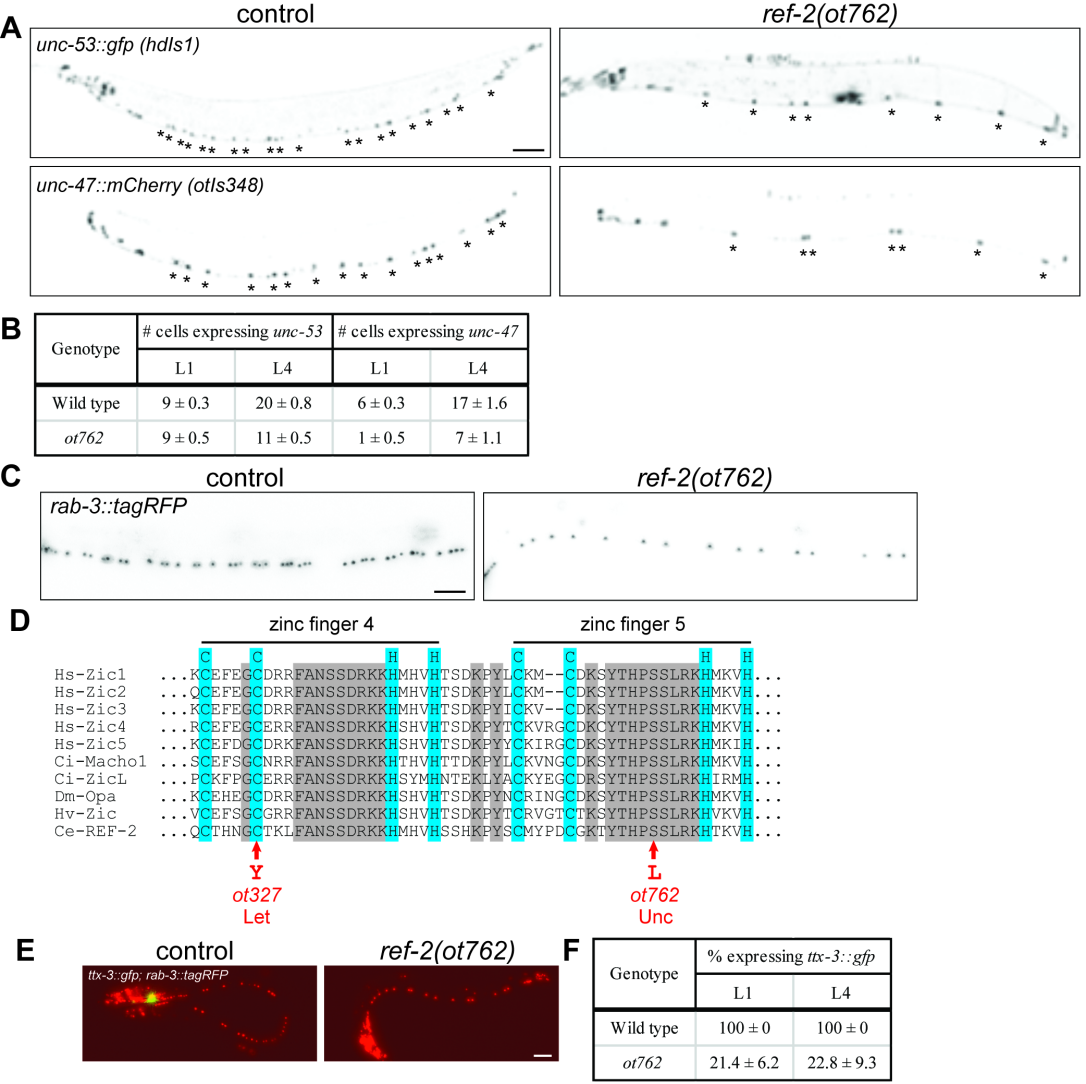
***ref-2(ot762)* affects neuron development.** (A) Fluorescent micrographs of *unc-53::gfp* (DA and AS) and *unc-47::mCherry* (DD and VD) in L4 wild-type control and *ot762* mutant animals, with ventral nerve cord motor neurons indicated by asterisks (scale bar 50µm in all panels). (B) Quantification of the number of VNC neurons expressing *unc-53::gfp* and *unc-47::mCherry* in controls and *ot762* mutants at L1 and L4 stages (mean ± standard deviation). (C) Confocal micrographs of *rab-3::tagRFP* (all VNC neurons) in L4 control and *ot762* mutant. The average number of *rab-3::tagRFP(+)* cellsin the central region of the VNC decreases from 42.3 to 21. (D) Amino acid sequence of *C. elegans* of the fourth and fifth Zn finger of REF-2 aligned with orthologs from other species and location of amino acid substitutions caused in *ot327* (Let = lethal phenotype) and *ot762 (*Unc = uncoordinated phenotype)alleles indicated below sequence. Hs = *Homo sapiens*, Ci = *Ciona intestinalis*, Dm – *Drosophila melanogaster*, Hv = *Hydra vulgaris* (Hv) , Ce = *C. elegans.* Blue indicates zinc coordinating cysteine and histidine residues, grey indicates complete conservation in species shown. (E) Fluorescent micrographs of *ttx-3::gfp* (AIY interneuron) in L1 control and *ot762* mutant. (F) Quantification of the percent of worms with *ttx-3::gfp* expression in AIY in controls and *ot762* mutants at the L1 and L4 stages (mean ± standard deviation). In all images, anterior is to the left.

## Description

To better understand motor neuron specification, we have conducted an EMS-induced mutant screen in which we sought to identify mutants with defects in the expression of an *unc-53::gfp* marker (*hdIs1* transgene), which is normally expressed in the cholinergic DA and AS motor neurons (Wacker *et al.*, 2003). As previously reported, this screen identified the zinc finger transcription factor *bnc-1* (Kerk *et al.*, 2017). Another allele identified from this screen is *ot762*. Unlike *bnc-1* mutant alleles, *ot762* mutant animals are Unc and Egl. *ot762* mutants show a decrease in the number of *unc-53::gfp* expressing neurons in the L4, but not L1 stage, indicating a loss of *unc-53::gfp* expression in the postembryonically generated AS neurons, but not the embryonically born DA neurons (Fig.1A,B). The lineal sister of the cholinergic AS neurons are the GABAergic VD motor neurons (Sulston, 1976). We find that an *unc-47::mCherry* marker (*otIs348*; (Gendrel *et al.*, 2016)) also fails to be expressed in postembryonically generated VD motor neurons of *ot762* mutants; in addition, there is also a reduction in the number of embryonically generated DD neurons (Fig.1A,B). Analysis of a panneuronal marker, *rab-3*, indicates a reduced number of neurons in the ventral nerve cord (Fig.1C), supporting the possibility that AS, DD and/or VD neurons may not be generated.

Using a combined polymorphic mapping and whole genome sequencing pipeline (Minevich *et al.*, 2012) we found that *ot762* animals harbor a missense mutation in the 5^th^ zinc finger domain of the highly conserved zinc finger transcription factor REF-2, called Zic1/2/3 in vertebrates and Opa in flies (Alper and Kenyon, 2002; Aruga, 2004) (Fig.1D). The mutation results in the substitution of a highly conserved serine to a leucine within the last Zn finger domain of REF-2. This specific amino acid position is predicted to contact DNA (Benos *et al.*, 2002). Previous analysis in *C. elegans* has shown that *ref-2* null mutants display an L1 larval lethal phenotype, possibly due to a disruption of the excretory system (Bertrand and Hobert, 2009; Bordet and Bertrand, 2018). However, while *ot762* animals are Unc (consistent with a function in motor neuron development), they do not die at L1. Therefore, *ot762* separates distinct functions of *ref-2* in different cell types. *ref-2* has also previously been shown to act upstream of the terminal selector *ttx-3* to control AIY interneuron specification (Bertrand and Hobert, 2009). We find that *ot762* animals display strong defects in *ttx-3::gfp* expression (Fig.1D,E), albeit at a somewhat lesser penetrance than the *ot327* null allele (Bertrand and Hobert, 2009).

*ref-2* is expressed in a number of neuroblasts, but not in the mature, adult nervous system (Bertrand and Hobert, 2009). Among the neuroblasts that express *ref-2* are the P neuroblasts (Alper and Kenyon, 2002; Bertrand and Hobert, 2009), which give rise to a number of motor neurons, including the AS and VD sister neurons (Sulston, 1976). Together with the *ref-2(ot762)* mutant phenotype that we describe here, as well as with P cell developmental defects described in *ref-2* mutants (Alper and Kenyon, 2002), this suggests that *ref-2* acts at some point in the P lineage to affect the differentiation of the AS and VD motor neurons. The phenotype that we observe in the AIY neurons of *ot762* is a reflection of the previously described function of *ref-2* in the neuroblast that generates the AIY neuron (Bertrand and Hobert, 2009). DD neuron differentiation defects observed in *ot762* mutants may be a reflection of *ref-2* function in embryonic neuroblasts akin to the function of *ref-2* in the AIY-generating neuroblast.

## Reagents

OH12225 *ref-2(ot762); hdIs1[unc-53::gfp]; otIs348[unc-47::mCherry]*
